# Evaluation of Bioactive Metabolites and Antioxidant-Rich Extracts of Amaranths with Possible Role in Pancreatic Lipase Interaction: In Silico and In Vitro Studies

**DOI:** 10.3390/metabo11100676

**Published:** 2021-09-30

**Authors:** Swati Chaturvedi, Promila Gupta

**Affiliations:** Agriculture Plant Biotechnology Laboratory (ARL-316), University School of Biotechnology, Guru Gobind Singh Indraprastha University, Dwarka, Delhi 110078, India; swati.chaturvedi87@gmail.com

**Keywords:** Amaranthaceae, antioxidants, fluorescence quenching, molecular docking, pancreatic lipase, phytochemicals, proximate contents, volatile metabolites

## Abstract

Fat/carbohydrate-rich diet consumption or elevated secretion of pancreatic lipase (PL) in pancreatic injury results in increased fat digestion and storage. Several metabolites in plant-based diets can help achieve the requirements of nutrition and fitness together. Presently, nutritional metabolites from *Amaranthus tricolor*, *A. viridis*, and *Achyranthes aspera* were assessed and predicted for daily intake. The volatile-metabolite profiling of their extracts using GC-MS revealed various antioxidant and bioactive components. The implication of these specialized components and antioxidant-rich extracts (EC_50_ free radical scavenging: 34.1 ± 1.5 to 166.3 ± 14.2 µg/mL; FRAP values: 12.1 ± 1.0 to 34.0 ± 2.0 µg Trolox Equivalent/mg) in lipolysis regulation by means of interaction with PL was checked by in silico docking (Betahistine and vitamins: ΔGbind −2.3 to −4.4 kcal/mol) and in vitro fluorescence quenching. Out of the various compounds and extracts tested, Betahistine, ATRA and AVLA showed better quenching the PL fluorescence. The identification of potential extracts as source of functional components contributing to nutrition and fat regulation can be improved through such study.

## 1. Introduction

Plants produce several chemicals other than the primary metabolites (nutrient components), which take care of their defense needs besides others. These include various phytochemicals such as phenolics, terpenoids, saponins, and alkaloids with functional and health-promoting properties. Consumption of green leafy vegetables (GLV) in various forms is not a new phenomenon across the globe and is trending now due to the above qualities [1]. Amaranthaceae is one such family of more than 60 species of annual or perennial plants, including herbs, vines, and shrubs which are rich in these phytoconstituents, vitamins, and other essential nutrients. They comprise economically important cultivated or wild food crops (vegetable and GLV: beet (*Beta vulgaris*), spinach (*Spinacea oleracea*), and Amaranthus (*A. tricolor* and *A. dubius*), pseudograins like *A. caudatus* and *A. hypochondriacus*, as well as Chenopodium spp., decorative plants (*A. caudatus* and *Celosia argentea*), and several detrimental weeds (*A. viridis*, *A. retroflexus*, A. hybridus, and *A. gracilis*) worldwide. Some of these have also been reported for their medicinal uses in diarrhea, diabetes, blood pressure, and antimicrobial activities despite growing in the wild [2,3,4,5,6,7].

Lack of physical activity and high-calorie intake result in metabolic disturbances which stimulate fat accumulation and oxidative stress in the body [8,9]. Pancreatic lipase (PL) is one of the crucial enzymes in fat metabolism. Under the influence of fat or carbohydrate-rich diets, it can contribute to the increase in visceral fat that may lead to obesity [10]. Additionally, excess free radicals can fuel up this fat deposition in the body. Increased lipid digestion due to excessive secretion of PL is also linked to the recent studies on COVID-19 induced pancreatic injury (pancreatitis) [11].

The antioxidant agents can alleviate oxidative stress reducing the risk of obesity [12]. Two drugs, orlistat (Xenical), the only drug approved by USFDA, and cetilistat (ATL-962) (under clinical trial) are used to regulate PL activity. These bind with PL catalytic residues causing covalent modification, further inhibiting its activity and lessen the absorption of fat from food [13,14,15].

Several recent studies have reported different plants and phytochemicals for functional efficacy as antioxidant and PL interacting agents [16,17,18]. Wild vegetables from Nepal have been found to be rich in antioxidant metabolites [19]. *Achyranthes aspera* L. extracts rich in phenolic acids possessed protein binding capacity [20], while *Cornus* sp. fruit extracts, rich in phenolic acids and anthocyanidins showed antioxidant and PL inhibition activities [21]. Various flavonols such as Kaempferol, Galangin, Quercetin, and Myricetin showed the PL binding mechanism by quenching its fluorescence [22]. Some studies were also conducted on the therapeutic efficacy of phytochemicals and pigments in *Amaranthus* spp. as their novel source, focusing on antioxidant and antiobesity activities by PL inhibition and regulation of metabolic syndrome [23,24]. For instance, piperidine and di-terpenes rich *B. vulgaris* extracts and its juice can improve stamina, counter oxidative stress and inhibit PL activity [25,26,27], while phenolics and tannins rich extracts of shade-dried leaves of *A. tricolor* L. and green morph *Amaranthus* leafy vegetable were effective against oxidants and obesity [28,29,30]. *A. aspera* L. seeds and leaves exhibited antiobesity potential targeting α-amylase and α-glucosidase activities [31]. Metabolite fingerprinting revealed the presence of essential fatty acids, phenolic acids, and their precursors in the extracts of *A. aspera* showing antioxidant activities [32]. Extracts of *A. viridis* leaves have the potential to treat hypercholesterolemia [33]. Therefore, the strategy of bioassay-guided identification of the functional metabolites and their precursors from different sources, is a quick and essential advancement to achieve nutrition-based immunity and fat regulation as well.

In the present study, three plants from the Amaranthaceae family (*A. aspera* L. (AA), *A. viridis* L. (AV), and a leafy vegetable, *A. tricolor* L. (AT), commonly known as devil’s horsewhip, slender amaranth, and red amaranth, respectively) were compared for their functional potential as a possible and good source of antioxidants and PL interacting agents. Proximate compositions and mineral contents in the powdered samples of their different parts (leaves: L; stems: S; and roots: R) were compared. Further, screening of their crude acetone and methanolic extracts was conducted for phytochemicals (total phenolic contents—TPC and total flavonoid contents—TFC) and antioxidant potential using the mimicking radicals. The presence of volatile metabolites was determined in three shortlisted extracts which showed good antioxidant activities, using gas chromatography and mass spectrometry (GC-MS). It was followed by fluorescence-based interaction and in silico studies of potential extracts and their components with PL, respectively. The oral bioavailability of the relevant compounds was also predicted based on Lipinski’s rule of five (Lipinski’s RO5).

## 2. Results and Discussion

### 2.1. Heavy Metals, Minerals, and Proximate Contents

The proximate compositions were assessed on the dry weight basis, and the values were expressed as percentages (Table 1). Moisture percentages were calculated on the processed samples considering fresh weight, which were further used for the proximate analysis. As expected, ATL had the highest and most significant moisture percentage compared to other samples (Table 1). The ash (inorganic residue equivalent) was 12.5% in seeds extracts of AA in a study [31] which was higher than in the tested AAL samples but lesser than in the AVL and ATL samples. Crude protein content (total nitrogen) was highest in the AVL sample, and it was higher than in the leaves and petiole of beetroots (24 and 13% dry weight, respectively) [25]. Fat contents were in traces. Carbohydrate analysis indicated that root parts had higher contents than the other parts with AAL, which had the highest percentage (Table 1). The moisture content of the ATL sample (ATL non-significant with AVL only) was most significant than other samples (*p* ≤ 0.05). For crude protein, AVL, and ash contents, ATS, AVR, and AAL were the most significant (*p* ≤ 0.05) samples (Table 1). 

In today’s environment, plants are at a high risk of becoming contaminated with heavy metals, and their entry into the food chain may be life-threatening; however, they can be a good source of essential minerals too. Therefore, an assessment of heavy metals toxicity, minerals, and nutrient status should be done before analyzing the nutraceutical potential of the plants. The analysis may also help improve the quality and nature of the soil and water from the location of samples collection. The results of heavy metals and minerals analyses of the powdered samples were reported in ppm based on sample dry weight. It showed the presence of essential minerals such as chromium (Cr), copper (Cu), iron (Fe), manganese (Mn), selenium (Se), and zinc (Zn), in adequate concentrations. These micronutrients may facilitate metabolism by their presence despite the minimal requirement (Table 1). Zn can neutralize the toxic effects of Cd, Zn along with Fe and Cu is essential in anemia and keeps central nervous system healthy, while Cr is essential for insulin activity and DNA transcription, etc. [34]. Heavy metals arsenic (As), cadmium (Cd), lead (Pb), mercury (Hg), and tin (Sn) were found in much lower concentrations (Table 1). World health organization (WHO) permissible limits for the daily intake of heavy metals are expressed in terms of provisional tolerable intake (PTI) and is calculated by body weight of an average adult and the recommended consumption of foodstuff per person per day (Table 2) [34]. The daily intake of these trace elements by Indians has been estimated considering the recommended dietary allowances (RDA) for GLV consumption, 100 g per day (Table 2). The values in Table 2 showed most of the samples having non-significant concentrations of micronutrients compared to RDA, thereby representing acceptable sources of essential nutrients.

### 2.2. Extraction and Preliminary Phytochemicals Screening

#### 2.2.1. Yield

It is crucial to isolate the components of interest from the plant materials for their detailed characterization [42]. Therefore, extraction of three different parts of AV, AT, and AA was done in solvents with different polarity to extract these phytoconstituents in their crude forms for further analysis. MeOH was determined as the better extractant than acetone as the percent yields of eight acetone extracts (AVLA-2.7 > ATLA-2.2 > AVSA-1.5 > ATRA = AASA-0.8 > AVRA-0.7 > AALA-0.5 > ATSA-0.2) were lesser than that of eight methanolic extracts (ATSM-13.7 > AVRM-11.5 > AVSM-10.9 > ATRM-8.6 > ATLM-5.4 > AVLM-5.2 > AASM = AALM-~2). 

#### 2.2.2. Estimation of TPC and TFC

**Figure 1:**
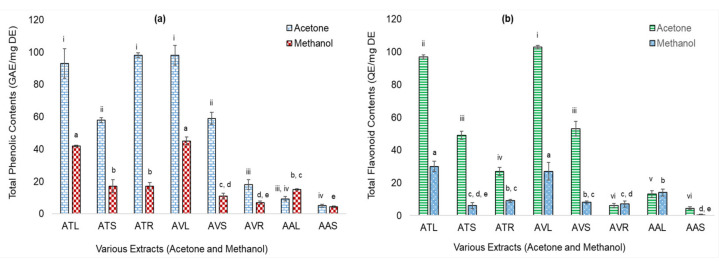
Total Phenolic Contents (TPC) (as microgram Gallic acid equivalents/milligram dry extracts: µg GAE/mg DE) and Total flavonoid contents (TFC) (as µg Quercetin equivalents/mg DE: µg QE/mg DE) of acetone and methanolic extracts of *Achyranthes* *aspera* L. (AA), *Amaranthus* *viridis* L. (AV), and *Amaranthus tricolor* L. (AT) plants parts. (**a**) TPC of extracts; (**b**) TFC extracts where, ATL = AT leaves; ATS = AT stems; ATR = AT roots; AVL = AV leaves; AVS = AV stems; AVR = AV roots; AAL = AA leaves; and AAS = AA stems. Values are significant at *p* ≤ 0.01 and ≤ 0.05 (Different alphabets and roman numerals on the bars, show statistical difference in values within the same group).

Eight extracts based on their higher TPC and TFC values were ranked in decreasing order and selected for antioxidant assays. TPC of acetone extracts of AV leaves, AT roots, and AT leaves were significantly higher (*p* ≤ 0.05) than the other five extracts (Figure 1a). TFC of acetone extracts of AV and AT leaves were significantly higher (*p* ≤ 0.05) than the other six extracts (Figure 1b). TPC values of all the acetone extracts were insignificant to each other whereas TFC of acetone extracts of leaves were significant (*p* ≤ 0.05) to that of the stem and root extracts. In methanolic extracts, both TPC and TFC of leaf extracts were significant (*p* ≤ 0.05) to that of stems and roots.

These values were significantly higher than the ethanolic extracts of AA seeds and equivalent to the methanolic extracts of its shade-dried aerial parts (TPC = 0.34 µg and TFC = 0.30 µg; TPC = 14.28 ± 0.24 µg, respectively) [31,32]. TPC values of acetone extracts of AV leaves and AV stems were also higher than the reported TPC of 50% methanolic extracts of AV leaves and stems (85.8 and 26.4 µg, respectively) [33] and that of beetroot leaves acetone and methanolic extracts (43 and 64 µg, respectively) [25]. The results indicated that in methanolic extracts, only the leaves of AV, AT, and AA were high in TPC and TFC. In acetone extracts, leaves of AV and AT were higher, followed by their stem extracts (five out of eight extracts were the leaves extracts). 

#### 2.2.3. Antioxidant Potential

**Figure 2:**
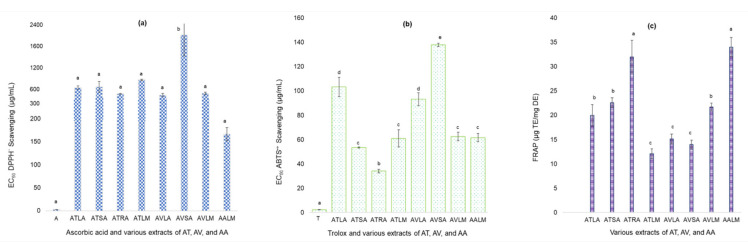
Antioxidant potential of extracts of *Achyranthes* *aspera* L. (AA), *Amaranthus* *viridis* L. (AV), and *Amaranthus tricolor* L. (AT). (**a**) Extracts vs. EC_50_ (µg/mL) in DPPH assays; (**b**) Extracts vs. EC_50_ (µg/mL) in ABTS assays; (**c**) FRAP values of extracts where, A = ascorbic acid; ATLA = acetone AT leaves; ATSA = acetone AT stems; ATRA = acetone AT roots; ATLM = methanolic AT leaves; AVLA = acetone AV leaves; AVSA = acetone AV stems; AVLM = methanolic AV leaves; AALM = methanolic AA leaves; T = trolox. Values are significant at *p* ≤ 0.01 and ≤ 0.05 (Different alphabets on the bars, show statistical difference within the same group).

Antioxidant properties of the selected eight extracts were evaluated by observing the scavenging of mimicking free radicals (decolorization of DPPH^•^ radical and conversion of ABTS^•+^ radical cation to ABTS) in DPPH and ABTS assays and the reducing potential in FRAP assay. The EC_50_ (µg extract/mL) of extracts were shown as the bar graphs (Figure 2a,b); however, the scavenging of DPPH^•^ and ABTS^•+^ radicals were concentration-dependent for all.

A lower EC_50_ value indicates the better potential of an extract for free radical scavenging than the comparative extracts. The EC_50_ of positive controls A and T were significantly (*p* ≤ 0.05) lower than the crude extracts in their respective assays (Figure 2a,b). EC_50_ of acetone extracts of AV stems extracts for DPPH^•^ scavenging was statistically significant (*p* ≤ 0.05) and higher than the other extracts. Methanolic extracts of AA leaves, acetone extracts of AT roots and AV leaves showed EC_50_ below 400 µg/mL. These values were much lower than those of the various extracts of AV leaves (in chloroform, MeOH, and aqueous) as reported in earlier studies [28], but higher and equivalent to that reported for methanolic extracts of leaves and roots of AA and GLV, respectively, by Rana et al., 2019 and Dasgupta and De, 2007 [43,44]. For ABTS^•+^ scavenging, EC_50_ of acetone extracts of AT and AV leaves were more significant than the other extracts but mutually non-significant (*p* ≤ 0.05). EC_50_ of acetone extracts of AT roots was the best with the lowest value. Figure S1 shows the concentration-dependent scavenging (in percent) of DPPH^•^ and ABTS^•+^ radicals at various concentrations of three potential extracts (AALM, ATRA, and AVLA). Reducing the potential of extracts in terms of TE (Figure 2b) showed that methanolic extracts of AA leaves and acetone extracts of AT roots extracts had the highest and non-significant (*p* ≤ 0.05) values. Acetone extracts of AV leaves and stems, and methanolic extracts of AT leaves had the lowest and non-significant (*p* ≤ 0.05) FRAP value (Figure 2b). These equivalents were higher than those reported in a previous study for AA, *A. ganjetica*, and AV (aqueous extracts: 2.4, 2.1, and 1.6; methanolic extracts: 2.2, 2.1, and 1.5 TE) [45]. 

### 2.3. Analyses GC-MS Chromatograms

The GC-MS chromatograms of AALM, ATRA, and AVLA extracts reported various compounds in the experimental run of 50 min for each. Different terpenes (mono-, di-), sterols, unsaturated fatty acids (such as ω-3 fatty acids), vitamins, alcohols, fatty amide, and their esters, precursors, and metabolites with variations in their area percentage were identified. Some of these were reported in earlier studies to effectively regulate various health conditions such as oxidative stress, obesity, anticancer, and antimicrobial potential, with a recent in silico study of being active against the SARS-CoV virus [46]. The relevant compounds based on the respective peak area percentages and reported nutraceutical values are listed in Table 3, which show that most of the components reported to be active in oxidative stress, have also been implicated as antiobesity agents. Therefore, the present study attempted the exploration of the antiobesity property of some antioxidants by the means of their interaction with lipase.

### 2.4. In Silico and In Vitro Binding with PL

#### 2.4.1. In Silico Interaction with PL, Drug-Likeness, and Bioavailability

**Figure 3:**
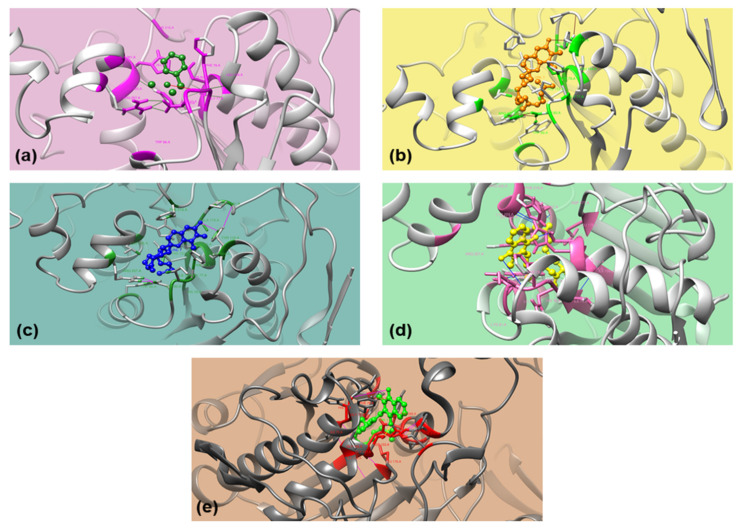
3D orientation of the docked conformations of 1ETH with ligands. (**a**) Betahistine; (**b**) α-Tocopherol; (**c**) γ-Tocopherol; (**d**) Tocopheryl acetate; and (**e**) Phytonadione.

The docking conformations were ranked in ascending ΔG_bind_ in obtained clusters and runs [74]. The best fit in 1ETH-ligand interaction was determined using the conformation having the lowest ΔG_bind_, K_i_, and H-bond in that 1ETH-ligand complex. Out of 7 ligands, betahistine (Figure 3a), α-tocopherol (Figure 3b), γ-tocopherol (Figure 3c), tocopheryl acetate (Figure 3d), and phytonadione (Figure 3e) were docked successfully with PPL (1ETH). The compounds failed to interact with 1ETH may have a different mode of action in PL inhibition and antiobesity activity. 

Betahistine, α-tocopherol, γ-tocopherol, phytonadione, and tocopheryl acetate interacted with PPL (1ETH) through H-bonds. Although tocopheryl acetate and phytonadione had higher ΔG_bind_, the two interacted very well with two residues, serine (Ser153) and phenylalanine (Phe78) of 1ETH (Figure 3d,e). Betahistine had the lowest ΔG_bind_ and bonded with one residue, Phe78, whereas α-tocopherol and γ-tocopherol bonded with tyrosine (Tyr115) through H-bonds and had equivalent ΔG_bind_ (Figure 3a). However, ΔG_bind_ of the above ligands were higher than that of Phytol, a diterpene (−5.33 kcal/mol) which have been shown for comparatively good interaction with 1ETH involving two residues, Tyr115 and Phe216 (2 H-bonds) [25].

PPL has intrinsic fluorescence attributed to phenylalanine (Phe), tryptophan (Trp), and tyrosine (Tyr) amino acid residues where the fluorescence intensity (FI) is mainly due to the Trp residue. The interaction of ligands with Phe and Tyr residues besides active site residue Ser indicated their role towards the change in intrinsic fluorescence of PPL; however, any of these ligands did not interact with Trp residue.

The comparison of the prediction scores for molecular and physicochemical properties of the compounds showed that betahistine followed all the parameters of Lipinski’s RO5, whereas the other 4 violated one parameter (Table 4). The radar plots of tocopheryl acetate, α-tocopherol, γ-tocopherol, and phytonadione crossed the pink zone as of betahistine. Hence, betahistine was more suitable for being considered a good candidate as an antiobesity agent with the optimal physicochemical requirements for oral bioavailability (the probability was 0.55 for each). 

#### 2.4.2. Effect on Intrinsic Fluorescence of PPL and Quenching Statistics

**Figure 4:**
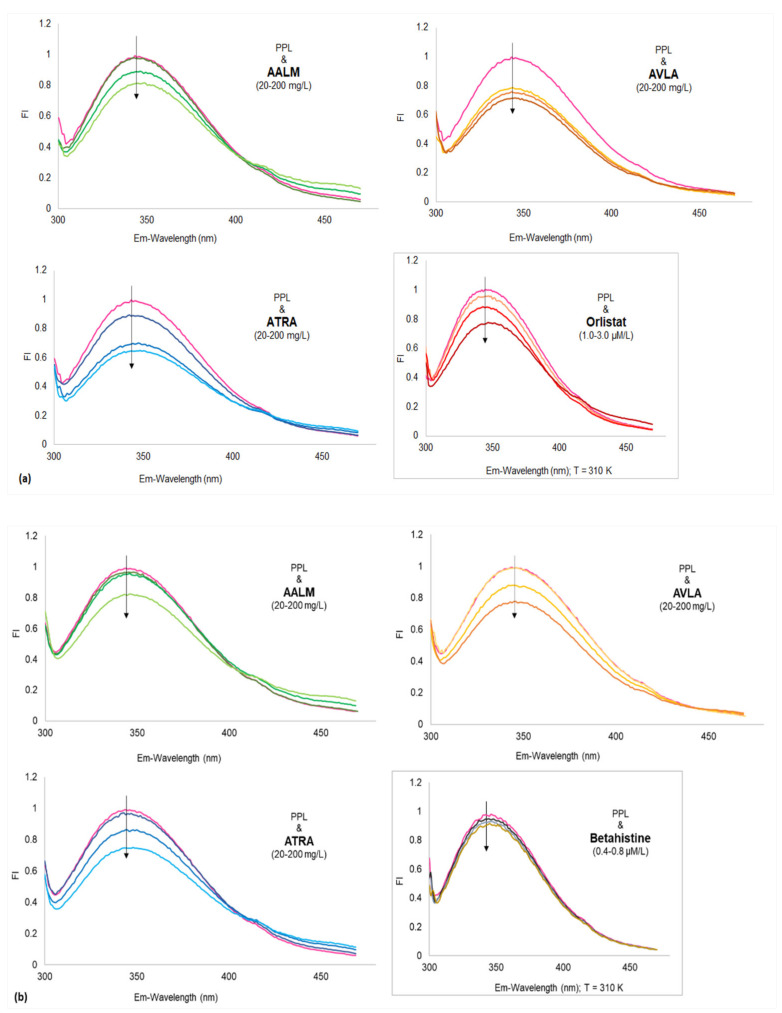

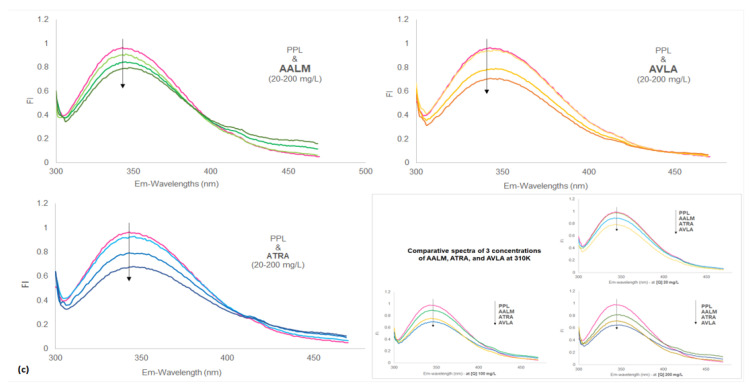
Fluorescence spectra of porcine pancreatic lipase (PPL) shows fluorescence quenching in the presence of extracts and compounds. Fluorescence intensities (FI) vs. wavelengths (λ nm) at different temperatures (**a**) 310; (**b**) 320; and (**c**) 330 K temperatures (PPL + extracts: 20, 100, and 200 mg/L); Insets show: PPL + orlistat: 1.0, 2.0, and 3.0 µM/L (**a**), PPL + betahistine: 0.4, 0.6, and 0.8 µM/L (**b**) and Comparative spectra of 3 concentrations of AALM, ATRA, and AVLA (**c**), at 310 K.

The x-ray structure of PPL (1ETH) from in silico study showed three out of seven Trp residue located near the active site in the N-terminal. The addition of a quencher decreases the intrinsic fluorescence of PPL when it interacts with Trp, Tyr, and Phe residues by changing the polarity of their microenvironment. In-silico analyses herein showed that besides interacting with Ser, Tyr, or Phe residue(s), none of the compounds interacted with Trp residue at tested concentrations resulting in a minor decrease in FI. However, the interaction of the crude extracts had a notable decrease in FI with a possibility for the presence of Trp interacting agents along with the other residues (Figure 4). FI vs. wavelength (λ nm) showed the interaction of various concentrations of AALM, ATRA, and AVLA extracts, betahistine hydrochloride, and orlistat with PPL where FI was decreasing with the increase in the test concentrations (Figure 4; T = 310, 320, 330 K).

No apparent red shifts (z) of the maximum emission of PPL fluorescence were observed by the samples at the tested concentrations. However, AALM and ATRA extracts caused a slight bathochromic shift with increasing concentrations, which decreased as the temperature increased (T: 310–330 K, z = 0.018–0.012). In contrast, the addition of AVLA extracts enhanced that minor shift with the increase in temperature (T: 310–330 K, z = 0.009–0.015), indicating more changes in the polarity of the microenvironment and quenching.

**Figure 5:**
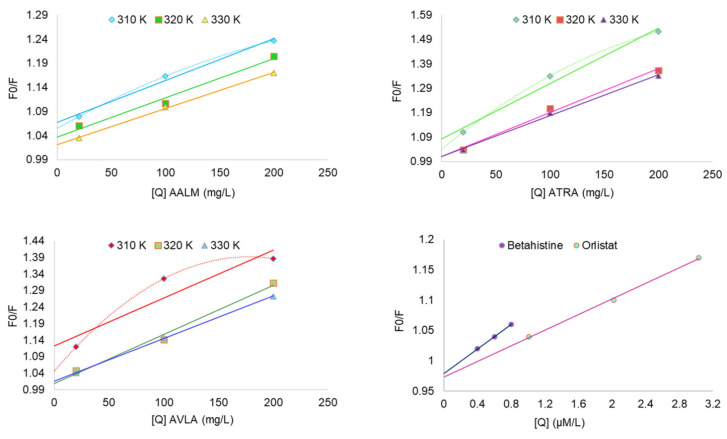
Kinetics of PPL fluorescence quenching: Stern-Volmer plots show F_0_/F vs. concentrations [Q].

The quenching mechanism was estimated by analyzing the Stern-Volmer’s plots of the extracts (Figure 5). The improved linearity of extracts’ graphs (Figure 5) plotting F0/F vs. [Q] at different temperatures also showed more accessibility of extracts to PPL as the temperature increased. The extracts used for fluorescence experiments were in their crude forms (mix of a variety of compounds with different affinity for PPL). Therefore, their degrees of fitness for apparent quenching were acceptable (ranging from 0.87 to 0.99) [75]. Table 5 reported the various parameters of binding affinity calculated from these plots for each extract and compound. Their quenching rate constant (K_q_) and binding constant (K_a_) were reported as Lg^−1^ and Lg^−1^s^−1^ only (Table 5), and therefore, the mechanism of quenching was not clear. The decrease in K_q_ with increasing temperature (310 to 330 K) showed that the static complex formation might initiate that quenching (Figure 5) [75,76]. On the other side, the fluorescence quenching by betahistine, and orlistat were clearly static as the magnitudes of K_q_ were of order 10^13^ which were much higher than the maximum collision quenching constant, i.e., 2.0 × 10^10^ LM^−1^s^−1^ [76].

**Figure 6:**
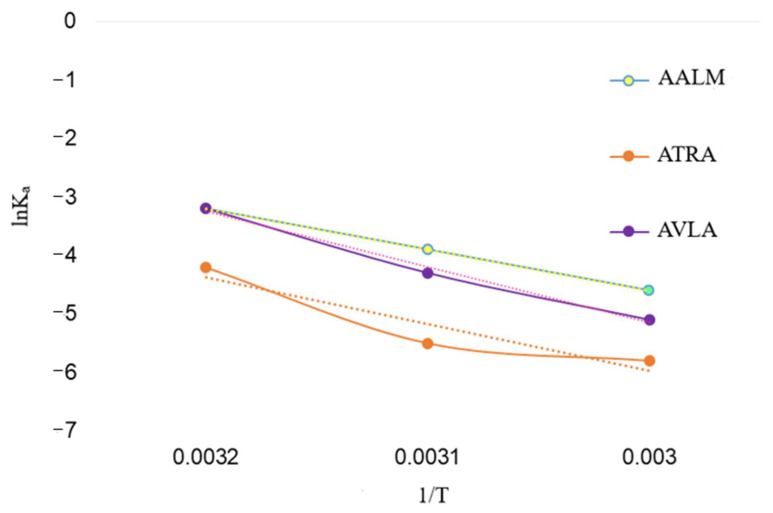
Kinetics of PPL fluorescence quenching: Plot shows relationship between interaction (K_a_) of extracts and temperature (lnK_a_ vs. 1/T).

K_a_ and number of binding sites per protein molecule (n) were calculated using the equation (4), where logK_a_ was directly proportional to n. The dependence of extracts interaction on temperature was presented in Figure 6 as lnK_a_ vs. 1/T; however, the thermodynamic parameters were not determined for the crude extracts.

The inverse relationship of K_a_ with temperature and negative free energy change (ΔG) represented the spontaneous (exothermic) interaction of PPL with extracts and compounds, respectively (Table 5). Additionally, the ΔG of PPL and compounds were in accordance with their high values of K_a_. The number of binding sites (n~1) showed that there was one binding site in PPL available to the extracts (Table 5) and, similarly more than one for the compounds. However, at temperature 320 K, *n* > 1 indicated that there might be a slight unfolding in PPL that gave more access to ATRA and AVLA to interact with PPL residues which were again decreased at 330 K and a decrease in K_a_, too.

### 2.5. Correlation and Significance

Correlation coefficients (r) of the extracts between different assays were determined. TPC of acetone and methanolic extracts were strongly correlated [77] with the TFC of the respective extracts (r = +0.829 and +0.946, respectively; *p* ≤ 0.01). Pearson’s correlation between TPC and TFC of all the extracts was strongly positive (r = +0.871; *p* ≤ 0.01). Along with the correlation, the statistical significance of the phytochemical contents and bioacivities of the best three extracts (AALM, ATRA, and AVLA) were also determined (Table 6).

There was a moderate correlation between TPC and TFC when the values of only three shortlisted extracts were compared (Table 6). Total phenolics were highly correlated with DPPH only and have no correlation with ABTS and FRAP whereas flavonoids were more correlated with ABTS and FRAP only. The correlation differences might be depending upon the forms of phenolics (bound or free) present in the extracts. The poor correlation between the three antioxidant assays were possibly due to their different reduction mechanisms. Therefore, radicals scavenging and reduction potential by the extracts indicated the presence of a suitable reducer for the assays, such as DPPH^•^ scavenging might be due to the presence phenolics in the extracts, while ABTS^•+^ scavenging and ferric reducing potential were due to the flavonoid contents. There might be another possibility that antioxidant activities of the extracts were not governed by TPC and TFC contents rather their precursors or the other volatile metabolites which have been reported as antioxidants in literature.

The correlation between Kq and Ka of these three extracts was not significant (r = +0.445). ΔG and K_a_ of compounds were strongly and significantly correlated (r = +0.988; *p* ≤ 0.01), while ΔG and K_q_ were moderately correlated (r = +0.708). The temperature showed a significant negative correlation with K_a_ (r = −0.385; *p* ≤ 0.05) and a non-significant negative correlation (−0.246) with K_q_.

## 3. Materials and Methods

### 3.1. Chemicals

Analytical and HPLC grade chemicals were used in the study. Aluminium chloride, copper sulfate, ferric chloride, Folin-Ciocalteu phenol (FCP) reagent, potassium phosphate salts, sodium acetate, sodium hydroxide, sodium phosphate salts, sodium sulfate, and solvents (acetone, dimethyl sulfoxide (DMSO), methanol (MeOH), and petroleum ether) were purchased from Sisco Research Laboratories Pvt. Ltd., Mumbai, India; potassium persulphate (PPS) was purchased from the Central Drug House Pvt. Ltd., Delhi, India; concentrated hydrochloric acid (HCl), 2,2-diphenyl-2-picrylhydrazyl (DPPH), gallic acid (GA), L-ascorbic acid (A), potassium acetate, quercetin (Q), sodium carbonate, and 2,4,6-tripyridyl-s-triazine (TPTZ) were purchased from Himedia Co., Mumbai, India; 2,2’-azino-bis-(3-ethylbenzthiazoline-6-sulphonic acid) diammonium salt (ABTS), 6-hydroxy-2,5,7,8-tetramethylchroman-2-carboxylic acid or trolox (T), orlistat, and porcine PL Type-II (PPL) were purchased from Sigma Aldrich Co., St. Louis, MO, USA; betahistine hydrochloride tablets were from Abbott India Limited; sterile syringe filters (cellulose acetate, 0.2 μm pore size, 25 mm diameter) were purchased from Axiva Sichem Pvt. Ltd., Sonepat, Haryana, India. 

### 3.2. Collection of Plants and Extracts Preparation

Samples (including parts, leaves: L; stem: S; and roots: R) of a GLV (AT) were obtained from a local market in Dwarka, Delhi, India, and the weed samples (AV and AA) were collected from the campus of Guru Gobind Singh Indraprastha University (GGSIPU), New Delhi, India. However, the roots of AA could not be procured to perform the experiments. Botanist, Prof. Promila Gupta (University School of Biotechnology-USBT, GGSIPU), taxonomically identified the plants and the voucher specimen (AA: IP-USBT/SC/01/15; AT: IP-USBT/SC/02/15; and AV: IP-USBT/SC/03/15) were submitted to the Agriculture Plant Biotechnology Laboratory, USBT, GGSIPU.

The plant parts were separated, washed, and dried using a hot-air oven (HICON, Grover Enterprises, New Delhi, India) at 35 °C overnight until the samples were completely dried. The samples were ground using a mixer-grinder (Model: Sarita AE-321, 18000 rpm, 500 Watt from Ankit Industries, New Delhi, India). Sequential extraction of the 8 powdered samples was done by maceration in an incubator shaker (Model: ISF-1-W, Adolf Kühner AG, Birsfelden (Basel), Switzerland). The samples were first extracted in 100% Acetone (1:20 *w*/*v*) followed by 100% MeOH (1:20 *w*/*v*) (for 24 h at 30 °C; filtered and the process repeated twice with each). The filtrates of plants samples with the respective solvents were pooled and dried in the oven to a constant weight, yielding 16 crude extracts. The extracts were stored at 4 °C (maximum of two months) until further use. The percent yields were calculated using the following equation (1):

Yield (%) = 100 (W_e_/W_p_) (1)

where, W_e_ = weight of the extract obtained and W_p_ = weight of the powder sample used for extraction.

### 3.3. Proximate Analysis

Proximate analyses were performed following the standard methods (official method, OM) of the Association of Official Analytical Chemists (AOAC, 2005) [78]. Approximately 1.0 g of the powdered plant samples (AAL, AAS, AVL, AVS, AVR, ATL, ATS, and ATR) were used to estimate total moisture (OM 930.04), ash (OM 930.05, using muffle furnace Model: KAA/956/C, Sri Krishnaa Enterprises, Secunderabad, Telangana, India), crude fat (OM 2003.05, extraction unit Model: E–816 HE, BÜCHI Labortechnik AG, Flawil, Switzerland), crude protein (OM 977.02 using Kjeldahl factor of 6.25, distillation unit Model: VAPODEST 200, C. Gerhardt GmbH & Co. KG, Königswinter, Nordrhein-Westfalen, Germany), and carbohydrate contents. The carbohydrate content was calculated as the difference from the other components analyzed. Heavy metals and minerals compositions were also determined to compare the nutritional status of the samples (OM 2015.01 (2018) using inductively coupled plasma mass spectrometry Model: 7800 ICP-MS, Agilent Technologies, Santa Clara, CA, USA) [79]. 

### 3.4. Stock Preparation and Assays

10 mg/mL stock solutions of all the crude extracts (acetone: AALA, AASA, ATLA, ATSA, ATRA, AVLA, AVSA, and AVRA; methanolic: AALM, AASM, ATLM, ATSM, ATRM, AVLM, AVSM, and AVRM) were prepared in solvents, acetone, DMSO, and MeOH to use in further experiments.

#### 3.4.1. Determination of TPC and TFC 

The DMSO stocks of 16 extracts were tested for the quantitative determination of TPC and TFC.

TPC: TPC were determined using FCPR method [80]. The method was optimized for the samples previously (R^2^ = 0.999) [25] and TPC were calculated as µg GA equivalents (E)/mg dry extracts (DE). Briefly (for each extract), ~100 µg extracts and distilled water (dH_2_O) were mixed to volume 1 mL in the test tube. 2 mL freshly prepared FCPR solution (1:10 *v*/*v* in dH_2_O) was added, and it was followed by adding 1000 mM sodium carbonate solution (1 mL). Finally, incubated at room temperature (RT ~24 ± 1 °C) for 15 min in the dark and the absorbance was measured at 765 nm using a spectrophotometer and microplate reader (Model: SpectraMax M2^e^, Molecular Devices, San Jose, CA, USA). 

TFC: A colorimetric assay was followed to determine TFC [81]. The method was optimized for the samples previously (R^2^ = 0.999) [25] and TFC (µg QE/mg DE) were calculated. Briefly, ~100 µg extracts were mixed with DMSO to volume 1 mL in the test tubes. 10% aluminium chloride (100 µL), and 1000 mM potassium acetate (100 µL) solutions were added. The reaction mixtures were incubated for 30 min (dark; RT) and the absorbance was measured at 415 nm.

#### 3.4.2. Total Antioxidant Potential

Eight extracts out of the total 16 acetone and methanolic crude extracts, which had the highest TPC and TFC values (ATLA, ATSA, ATRA, AVLA, and AVSA and AALM, ATLM, and AVLM), were tested for their total antioxidant potential using free radicals scavenging and ferric reducing antioxidant power (FRAP) assay. DPPH^•^ and ABTS^•+^ scavenging activities of extracts were optimized [25] using 5 different concentrations according to the methods given by Kedare and Singh (2011) and Ungurianu et al., 2019, respectively, with slight modifications [82,83] and the effective concentration_50_ (EC_50_) were calculated for each extract. The reducing potential of the extracts were determined using FRAP assay as given by Benzie and Strain, 1996 [84] and the values were expressed as µg TE/mg DE (R^2^ = 0.999).

DPPH assay: Briefly, 100 mM DPPH solution was prepared in MeOH and 100 µL extracts were mixed with 900 µL DPPH. Reaction mixtures were incubated for 30 min (dark; RT). The absorbance was measured at 517 nm against the MeOH blank. 

ABTS assay: Briefly, 7 mM ABTS and 2.45 mM PPS were mixed in 100 mM sodium phosphate buffer (pH 7.4) to prepare ABTS solution and incubated in the dark for ~16 h at RT to generate enough ABTS^•+^ radicals. The solution was diluted with buffer until an absorbance of 0.700 ± 0.02 was obtained at 734 nm. 10 µL extracts were mixed with 990 µL ABTS^•+^ solution and incubated for 5 min (dark; RT). The absorbance was measured at 734 nm against the buffer blank. 

AA and T were the positive controls in DPPH and ABTS assays, respectively. DMSO without the extracts was the negative control and the percentage scavenging were calculated using the equation (2) below:

Scavenging (%) = 100 (AC − AT/AC)(2)

where, AC = absorbance of the negative control and AT = absorbance of the treated sample.

FRAP assay: Briefly, fresh FRAP reagent was prepared mixing 20 mM ferric chloride, 10 mM TPTZ (prepared in 40 mM HCl) in 200 mM sodium acetate buffer (pH 3.6) in a ratio *v*/*v*/*v* 1:1:10. The extracts (~100 µg) were mixed with 900 µL FRAP reagent and incubated for 4 min (37 °C; dark). The absorbance was measured at 593 nm against the buffer blank.

### 3.5. *Volatile* Metabolite Profiling

The stock solutions (concentration 10 mg/mL) of the selected crude extracts (AALM, ATRA, and AVLA), prepared in Acetone and MeOH were filtered through the syringe filters. The samples were analyzed on GC-MS (Model: GCMS-QP2010 Ultra, Shimadzu Corp., Nakagyo-ku, Kyoto, Japan) at the Advanced Instrumentation Research Facility, Jawaharlal Nehru University, New Delhi, India. Rtx-5MS column (Crossbond 5% diphenyl/95% dimethyl polysiloxane, capillary dimensions 30 m × 0.25 mm ID × 0.25 μm df) was used to check the presence of volatile metabolites in the samples. The screened peaks were used to identify the compound using the NIST’14 web book library and Wiley 08 library. The programming for the oven temperature and mass spectrometer were the same as used in a previous study [25]. In brief:

Oven temperature programming: Initially, the temperature was set 100 °C for 3 min and then increased to 250 °C with a rate 5 °C/min for 2 min. After that, the temperature was set to 300 °C at a rate of 15 °C/min and kept steady for 25 min.

MS conditions: For an electron ionization mass spectrum the temperature of the ion source was set to 230 °C with the solvent cut time 4.5 min. The temperature of injection port was 260 °C, coupled with 6 µL washing volume and 270 °C interface temperature and the mass scan (*m*/*z*)-40-650. Helium (1.21 mL/min) was used as the carrier gas with a split ratio of 1:10. The total GC-MS running time was about 50 min. 

### 3.6. Interactions with PL 

#### 3.6.1. Molecular Docking and Drug-Likeness of the Selected Compounds

Seven compounds (6 extracts components + 1 synthetic form of a ligand) were selected for molecular docking with PL. These were betahistine, α-tocopherol, γ-tocopherol, vitamin K1 (phytonadione), adamantane, pentadecane, and tocopheryl acetate (a stable form of vitamin E), based on the GC-MS identification. These compounds are used as therapeutics, nutritional supplements, and potent antioxidants along with their reported potential in obesity regulation. However, their interaction with PL is not determined as a possible antiobesity mechanism [56,57,63,64,72,73]. Successfully docked 5 ligands (betahistine, α-tocopherol, γ-tocopherol, phytonadione, and tocopheryl acetate) were further analyzed for interaction with 1ETH in terms of, presence of H-bond(s), drug-likeness, etc., which led to in vitro PL interaction study. Molecular docking was done in the following steps:

Preparation of ligand and protein: The structures of the ligand (compounds) were prepared using ChemSketch (source: https://www.acdlabs.com, accessed on: 10 April 2021) and saved in MDL.mol format and converted to the .pdb format using Open Babel V3.1.1. 1ETH, the PDB format of the x-ray crystal structure of PPL (2.8 A resolution) was picked-up from RCSB-protein data bank (source: http://www.rcsb.org/, accessed on: 10 April 2021) and the attached heteroatoms were removed [85].

Energy minimization: Energy minimization of 1ETH was done using SPDBV software by removing the water molecules. The coordinates added in the process of energy minimization were removed and the prepared protein was used for docking.

Molecular docking: Autodock 4.2 (Scripps Research, La Jolla, CA, USA) was used for docking PPL (1ETH) with the ligands. First, the polar hydrogens, charges were added to the processed 1ETH and saved in .pdbqt format along with the ligand. A grid box was given the coordinates for one of the amino acid residues of the catalytic site of three amino acids residues, Ser153, Asp177, and His264 (i.e., Ser153). The protein was kept in the rigid frame, whereas the ligands were flexible. The default 10 runs were given with the various docking conformations using Lamarckian Genetic Algorithm (source: http://autodock.scripps.edu/faqs-help/manual/autodock-4-2-user-guide, accessed on: 10 April 2021). Autogrid and Autodock were run to prepare their interaction in .gpf and .dpf formats, respectively, and then converted to their respective .glg and .dlg formats. 

Analysis of interaction: The interaction strength of a enzyme-ligand complex is based on their docking scores (binding free energy (ΔGbind), inhibitory constant (Ki), and the presence and number of hydrogen bonds (H-bond)). The docking scores were obtained from the final .pdb file of 1ETH, and the interactions were observed using UCSF-Chimera. 

SwissADME (ADME: absorption, distribution, metabolism, and excretion), and Lipinski’s RO5 were used to predict the drug-likeness and oral bioavailability of the compounds. It gave the prediction scores analyzing their structural, molecular, and physicochemical properties (an online software: http://www.swissadme.ch/, accessed on 10 April 2021) [86]. A compound can be stated for its use as a drug if it follows Lipinski’s rule, according to which the molecular weight of the compound should be ≤ 500 g/mol, lipophilicity (log *p*) should be ≤ 4.15, the number of H-bond donors (HBD) and acceptors (HBA) in that compound should be ≤ 5 and ≤ 10, respectively, and violation(s) among the first 4 rules should be zero [86].

#### 3.6.2. Effect on Intrinsic Fluorescence of PPL

The interaction of extracts (AALM, ATRA, and AVLA), betahistine, and orlistat with PPL were compared by studying the change in the intrinsic fluorescence of PPL [87]. Briefly, 2 µL PPL (40 mg/mL) was mixed with 4 µL test samples (concentrations were: extracts 10, 100, and 200 mg/L; orlistat 1.0, 2.0, and 3.0 µM/L in DMSO; betahistine hydrochloride 0.4, 0.6, and 0.8 µM/L in buffer) at 310 K in 100 mM potassium phosphate buffer (pH 7.2) in total 200 µL reaction mixture. After equilibration for 5 min, the fluorescence spectra (300–480 nm) were obtained at an excitation wavelength of 278 nm using the microplate reader. DMSO ≤ 2% of the reaction mixture, was used as the negative control and orlistat was the positive control. Similar reactions were also done for crude extracts at 320 and 330 K temperatures. The quenching constant (K_sv_), quenching rate constant or bimolecular quenching constant (K_q_), binding constant (K_a_), and the number of binding sites (n) were determined using the Stern-Volmer kinetics (equations 3 and 4) [88,89]:

F_0_/F = 1 + K_sv_[Q] = 1 + K_q_τ_0_[Q] (3)

LogF_0_ – F/ F = LogK_a_ + nlogQ (4)

ΔG = −RTlnK_a_ (5)

where, F_0_ and F = fluorescence intensities of PPL in the absence and presence of extracts/compounds, respectively, [Q] = concentrations of extracts/compounds, τ_0_ = constant of the lifetime of the fluorophore (10^−8^ s), ΔG = free energy of the system, T = temperature of the system (Kelvin), R = the gas constant of 8.31 J (mol K)^−1^.

### 3.7. Statistical Analysis

The statistical analyses were done using IBM^®^ SPSS V25.0 software (Armonk, NY, USA), and the results were presented as mean ± standard deviation (SD) of three independent experiments (*n* = 3). Pearson’s correlation between TPC/TFC, antioxidant, and PPL interaction activity of the potential extracts was determined. Further, the data were analyzed using one-way ANOVA and Tukey’s post-hoc test of significance. The differences were considered significant at *p* ≤ 0.01 and *p* ≤ 0.05.

## 4. Conclusions

This study presented various extracts of *A. aspera, A. viridis, and A. tricolor* as noteworthy and comparable sources of the antioxidant agents and components with lipase binding potential, especially the leaves. The results suggested that incorporation of leaves and roots in the diet may regulate lipid metabolism and oxidative stress more efficiently.

Further, vitamin E, K1, and betahistine interacted well with lipase. The quenching of PL fluorescence by betahistine and such a prevalent phytoconstituents like vitamin and their precursor may encourage the strategy of time- and cost-effective reprofiling of plant-based bioactives. It may help selection of the specialized metabolites to design foods for fat regulation, realizing new challenges of having an option of a prime source of drug or food in need and other possibilities of their implications in nutraceutical industries as well.

However, the present study also proposes the substantial studies to be done with necessity, such as characterization of other non-volatile bioactive components in the potential extracts as well as the study of lipase inhibition with these metabolites to validate the mechanism showed herein. 

## Figures and Tables

**Table 1:** Proximate contents in the processed samples.

Proximate Composition		Samples							
		AAL	AAS	AVL	AVS	AVR	ATL	ATS	ATR
Moisture	(%)	8.23 ± 0.21 ^b^	n.d.^1^	8.43 ± 0.46 ^b^	7.08 ± 0.46 ^c^	7.25 ± 0.40 ^c^	9.51 ± 0.56 *^,a^	7.47 ± 0.27 ^b,c^	8.06 ± 0.10 ^b,c^
Ash		6.97 ± 0.13 *^,a^		15.73 ± 0.07 ^e^	17.67 ± 0.1 ^d^	11.9 ± 0.10 *^,b^	17.14 ± 0.06 ^d^	26.71 ± 0.11 *^,c^	15.61 ± 0.65 ^e^
Crude fat		0.02 ± 0.00		2.12 ± 0.24 *^,a^	0.06 ± 0.03	0.78 ± 0.04	0.089 ± 0.01	1.39 ± 0.21 *^,b^	0.54 ± 0.10
Crude protein		10.19 ± 0.66		28.31 ± 1.25 *,^a^	8.83 ± 0.37	6.73 ± 0.53	17.36 ± 0.58	17.05 ± 0.28	7.41 ± 0.68
Carbohydrate		75.28 ± 0.80 ^b^		45.30 ± 1.7 ^d^	66.34 ± 0.9 ^c^	73.33 ± 0.50 ^b^	65.83 ± 1.10 ^c^	47.33 ± 0.30 ^d^	58.10 ± 1.20 *^,a^
Heavy metals (Conc. in ppm)	As	0.15 ± 0.00	n.d.	0.1 ± 0.00 *^,a^	0.00	0.03 ± 0.00 *^,b^	0.00	0.00	0.16 ± 0.00
	Cd	0.2 ± 0.0		0.21 ± 0.0	0.09 ± 0.0	0.1 ± 0.0	0.26 ± 0.0 *	0.08 ± 0.0	0.11 ± 0.0
	Pb	n.d.		0.6 ± 0.1 ^b^	0.2 ± 0.0 ^d^	0.7 ± 0.0 *^,b^	0.4 ± 0.0 *^,a^	0.2 ± 0.0 ^d^	0.6 ± 0.0 ^b^
	Hg	0.06 ± 0.0 *		0.03 ± 0.0	0.03 ± 0.0	0.04 ± 0.0	0.03 ± 0.0	0.02 ± 0.0	0.02 ± 0.0
	Sn	0.2 ± 0.0 *		0.08 ± 0.0	0.03 ± 0.0	0.1 ± 0.0	0.04 ± 0.0	0.08 ± 0.0	0.01 ± 0.0
Essential minerals (Conc. in ppm)	Cr	0.5 ± 0.1	n.d.	0.5 ± 0.0	0.5 ± 0.0	0.5 ± 0.1	0.6 ± 0.1	0.5 ± 0.1	0.9 ± 0.0 *
	Cu	10 ± 0.4 ^a^		10.5 ± 0.4 ^a^	6 ± 0.5	5.3 ± 0.5	6.1 ± 0.4	4 ± 0.6	4.3 ± 0.0
	Fe	415 ± 51.1 ^a^		392.4 ± 42 ^a^	60.6 ± 17.3 ^c^	235 ± 22.4 ^b^	223.5 ± 4 ^b^	82.7 ± 3.8 ^c^	169.8 ± 6.8 ^b^
	Mn	69.8 ± 16.1 *^,a^		48.5 ± 2.7 ^b^	11.7 ± 1.2	19.8 ± 2.3	33.2 ± 3 ^b^	13.2 ± 1	n.d.
	Se	0.2 ± 0.0 ^a^		0.3 ± 0.1 ^a^	0.2 ± 0.1 ^a^	0.1 ± 0.0	0.04 ± 0.0	0.01 ± 0.0	0.02 ± 0.0
	Zn	30 ± 1.5		42.7 ± 4 ^a^	37.6 ± 4.7 ^a^	28.7 ± 6.4	38 ± 3.2 ^a^	20.2 ± 6	22.5 ± 1.6

^1^ n.d. Not done. Superscript alphabets (a–e) show significant values within the group at *p* ≤ 0.05. * Most significant value(s) within the group.

**Table 2:** Estimation ^1^ of daily intake of heavy metals and micronutrients by Indians (RDA for GLV consumption, 100 g/person ^2^/day).

Daily Intake (µg/Day)		Samples									
		AAL	AAS	AVL	AVS	AVR	ATL	ATS	ATR	FOA/WHO ^3^ (PTI)	RDA/TUL by NIN and FSSAI, India
Heavy metals	As	15 ^b^	n.a.	10 *^,c^	-	3*^,d^	-	-	16 ^b^	3.0 µg/kg body weight (bw) (PMTDI) [35]	1.1 *^,a^ mg/kg
	Cd	20 ^b^		21 ^b^	9 ^c^	10 ^c^	26 *^,a^	8^c^	11 ^c^	25 µg/kg bw (PTMI) [36]	0.2 ^b^ mg/kg
	Cu	1000 ^b^		1050 ^b^	600	530	610	400	430	0.5 mg/kg bw (PMTDI) [37]	1.7 *^,a^ mg/day
	Pb	N.A.		60 ^b^	20 ^d^	70 ^a^	40 ^c^	20 ^d^	60 ^b^	3.0 µg/kg bw (PMTDI) [36]	0.3 ^d^ mg/kg
	Hg	6 ^b^		3	3	4	3	2	2	4 µg/kg bw (PTWI) [35]	1.0 ^a^ mg/kg
	Sn	20 *^,b^		8 ^d^	3 ^e^	10 ^c^	4 ^e^	8 ^c,d^	1 ^e^	3.3 mg/kg bw (PMTDI) [38]	250 *^,a^ mg/kg
Essential minerals/ Micronutrients	Cr	46 ^b,c^	n.a.	50 ^b,c^	47 ^b,c^	48 ^b,c^	61 ^b^	58 ^b,c^	91 *^,a^	~0.037 ^b,c^ mg/day [39]	0.05 ^b^ mg/day
	Fe	41,500 ^b^		39,240 ^b^	6060 ^d^	23,500 ^c^	22,350 ^c^	8270 ^d^	16,980 ^c^	60 *^,a^ mg/day (Max) [40]	45 ^b^ mg/day
	Mn	6980 ^a^		4850 ^b^	1170	1980	3320 ^b^	1320	N.A.	8.3 ^a^ mg/day (Max) [39]	4 ^b^ mg/day
	Se	20 ^c^		30 ^b^	20 ^c^	10 ^d^	4 ^d^	1 ^d^	2 ^d^	~0.035 ^a,b^ mg/day [41]	0.04 *^,a^ mg/day
	Zn	3000		4270	3760	2870	3800	2020	2250	45 *^,b^ mg/day (Max) [37]	40 *^,a^ mg/day

^1^ Based on, A Short Report on Nutrient Requirements for Indians-RDA and EAR. ICMR/NIN Expert Group. 2020. ^2^ Man/Woman (adult with mean weight: 65/55 kg, respectively; Sedentary/Moderately/Highly Active). ^3^ Source: Joint FAO/WHO Expert Committee of Food Additives (JECFA), 2020. Safety evaluation of certain food additives: Updates. In: 89th meeting, virtual meeting, June 1–2, 2020. * Most significant value(s) within the group. Superscript alphabets (a–e) show significant values within the group at *p* ≤ 0.05. EAR: Estimated Average Requirement; FSSAI: Food Safety and Standards Authority of India; ICMR: Indian Council of Medical Research; FAO: Food and Agriculture Organization; n.a.: not applicable; NIN: National Institute of Nutrition; PTI: Provisional tolerable intake; PMTDI: Provisional maxi-mum tolerable daily intake; PTMI: Provisional tolerable monthly intake; PTWI: Provisional tolerable weekly intake; RDA: Recommended Dietary Allowances; TUL: Tolerable Upper Limit; WHO: World Health Organization.

**Table 3:** Biologically active compounds identified in GC-MS analyses of AALM, ATRA, and AVLA extracts.

Compound	Peak Area (%)	Molecular Weight (g/mol)	Biological Activity
AALM			
2,4-Di-tert-butylphenol and derivative	32.2	262	Antifungal; Antioxidant [47]; One of the indicators of gut microbiota balance in COVID-19 infection [48].
Adamantane derivative	30.1	164	Anti-neurodegenerative [49]; Antiobesity [50]; Antioxidant [51]; Antiviral [52].
Dioctyl phthalate (DEHP)	14.2	390	Antibacterial [53]; Anticancer; Antioxidant [54].
α-Tocopherol (Vitamin E form)	11.4	430	Anticancer [55]; Antiobesity and reduce lipid peroxidation; Antioxidant [56,57]; Regulation of immune function and inhibition of platelet aggregation [58].
Linalool derivative	7.53	168	Anticonvulsive [59]; Anticancer [60].
ATRA			
2,4-Di-tert-butylphenol and derivative	40.2	262	Antifungal; Antioxidant [47]; One of the indicators of gut microbiota balance in COVID-19 infection [48].
Pentadecane	12.5	212	Anticancer; Antidiabetic; Antiobesity; Antioxidant (a volatile composition of various bioactive plant extracts) [61].
Progesterone metabolite	11.7	315	Covid-19, and Anti-inflammatory [62].
Dioctyl phthalate (DEHP)	9.2	390	Antibacterial [53]; Anticancer; Antioxidant [54].
Betahistine dimer	7.9	241	Treatment of vertigo [63]; Antiobesity [64].
AVLA			
Phytol	23.43	296	Precursor for synthetic forms of vitamin E and K1 [46]; Antidiabetic; Antiobesity [65,66].
Chondrillasterol	13.2	412	α-glucosidase inhibitor [67]; Anti-ulcerogenic [68]; In-silico inhibitor SARS-CoV-2 [69].
Linolenic acid	9.8	278	Anti-inflammatory; Antiobesity [70].
Neophytadiene	9.5	278	Analgesic; Antipyretic; Anti-inflammatory; Antimicrobial; Antioxidant [71].
γ-Tocopherols (Vitamin E form)	3.8	416	Anticancer [55]; Antiobesity and reduce lipid peroxidation, Antioxidant [56,57]; Regulation of immune function and inhibition of platelet aggregation [58].
Phytonadione (Vitamin K1)	0.7	450	Antiobesity; Antioxidant [72,73].

**Table 4:** Docking and bioavailability scores, and drug-likeness of the docked compounds.

	Docking Scores				Lipinski-Type Properties					Bioavailability Radar ^1^
Compound	ΔGbind (kcal/mol)	H-bond between 1ETH residue-ligand	Residue(s)	Ki (mM)	LogP	HBD	HBA	Violation of Rule	Drug likeness	Pink zone shows optimal physicochemical range
**Betahistine** **136.2 g/mol**	−4.39	1	Phe78	0.61	0.55	1	2	0	Yes	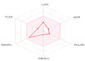
**Tocopheryl acetate** **472.7 g/mol**	+173.35	2	Phe78 and Ser153	-	6.36	0	3	1	Yes	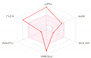
**α-Tocopherol** **430.7 g/mol**	−2.64	1	Tyr115	11.6	6.14	1	2	1	Yes	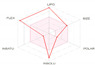
**γ-Tocopherol** **416.7 g/mol**	−2.28	1	Tyr115	21.3	5.94	1	2	1	Yes	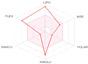
**Phytonadione** **450.7 g/mol**	255.24	2	Phe78 and Ser153	-		0	2	1	Yes	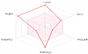

^1^ Parameters to estimate the oral bioavailability of each compound where LIPO indicates lipophilicity; POLAR indicates polarity; INSOLU indicates insolubility; INSATU indicates unsaturation; FLEX indicates flexibility.

**Table 5:** Summary of fluorescence quenching using Stern-Volmer’s kinetic parameters.

Kinetic Parameters (in Column) and Extracts/Compounds (in Rows)	Temperature (K)	Ksv (Lg^−1^) or (×10^4^ LM^−1^)	Kq (×108 Lg^−1s−1^) or (×10^13^ LM-1S^−1^)	Ka (Lg^−1^) or (× 10^4^ LM^−1^)	*n*	R2	ΔG (kJM^−1^)
**AALM (g/L)**	310	0.90 ± 0.21	0.90 ± 0.21 ^b^	0.04 ± 0.01 ^c^	0.91 ± 0.63	0.99	n.a. ^1^
	320	0.80 ± 0.14	0.80 ± 0.14	0.02 ± 0.03	0.58 ± 0.23	0.79	
	330	0.73 ± 0.12	0.80 ± 0.14	0.01 ± 0.004	0.70 ± 0.12	0.99	
**ATRA (g/L)**	310	2.30 ± 0.00	2.30 ± 0.00 ^c^	0.015 ± 0.01 ^c^	0.68 ± 0.05	0.99	n.a.
	320	1.80 ± 0.17	1.80 ± 0.17	0.004 ± 0.01	1.25 ± 0.57	0.99	
	330	1.67 ± 0.40	1.67 ± 0.40	0.003 ± 0.00	1.05 ± 0.20	0.99	
**AVLA (g/L)**	310	1.47 ± 0.60	1.50 ± 0.60 ^d^	0.041 ± 0.05 ^c^	0.66 ± 0.46	0.97	n.a.
	320	1.50 ± 0.16	1.50 ± 0.16	0.01 ± 0.02	1.46 ± 1.02	0.99	
	330	1.30 ± 0.56	1.30 ± 0.56	0.01 ± 0.01	0.89 ± 0.45	0.99	
**Betahistine (M/L)**	310	10.10 ± 0.60	1.01 ± 0.06 ^a^	8.91 ± 0.11 ^a^	1.67 ± 0.42	0.99	−29.36 ± 0.05 ^a^
**Orlistat (M/L)**	310	6.63 ± 4.90	0.66 ± 0.49 ^a^	3.91 ± 0.18 ^b^	1.21 ± 0.44	0.99	−27.24 ± 0.11 ^b^

^1^ n.a. Not applicable. Superscript alphabets (a–d) show significant values within the group at *p* ≤ 0.05.

**Table 6:** Pearson’s correlation coefficients (r) ^1^ between the assays.

Parameters	TPC	TFC	DPPH	ABTS	FRAP	Kq	Ka
**TPC**	-	+0.62	+0.95 **	+0.05	−0.57	+0.68 *	−0.12
**TFC**	-	-	+0.46	+0.80**	−0.90 **	+0.01	+0.13
**DPPH**	-	-	-	−0.09	−0.43	+0.84 **	−0.29
**ABTS**	-	-	-	-	−0.81 **	−0.45	+0.17
**FRAP**	-	-	-	-	-	+0.01	−0.12
**Kq**	-	-	-	-	-	-	−0.59

^1^ Correlation between the assays (DPPH and ABTS; FRAP (TE); PPL fluorescence parameters—K_q_ and K_a_) done with the best extracts (AALM, ATRA, and AVLA). The values show negative or positive correlation (0.9–0.8 for Strong, 0.7–0.6 for Moderate, 0.5–0.3 for Fair, 0.2–0.1 for Poor correlation). * Significant at *p* ≤ 0.05 and ** highly significant at *p* ≤ 0.01.

## Data Availability

Data is contained within the article and a Supplementary Figure S1.

## Supplementary Materials

The following are available online at https://www.mdpi.com/article/10.3390/metabo11100676/s1, Figure S1. Bar graphs show concentration-dependent free radical scavenging. Concentration (µg/mL) vs. % scavenging by 3 potential extracts: In DPPH assay (**a**) AALM (methanolic AA leaves), (**b**) ATRA (acetone AT roots), and (**c**) AVLA (acetone AV leaves) extracts; In ABTS assay (**d**) AALM, (**e**) ATRA, and (**f**) AVLA extracts.
