# Supplementary material for: Evaluation of Bioactive Metabolites and Antioxidant-Rich Extracts of Amaranths with Possible Role in Pancreatic Lipase Interaction: In Silico and In Vitro Studies
Source: Metabolites. 2021 Sep 30;11(10):676. doi: 10.3390/metabo11100676 (PMC8539516; doi:10.3390/metabo11100676)
Supplement: Supplementary file 1 [file metabolites-11-00676-s001.zip › metabolites-1388189-supplementary.pdf]

Supplementary material

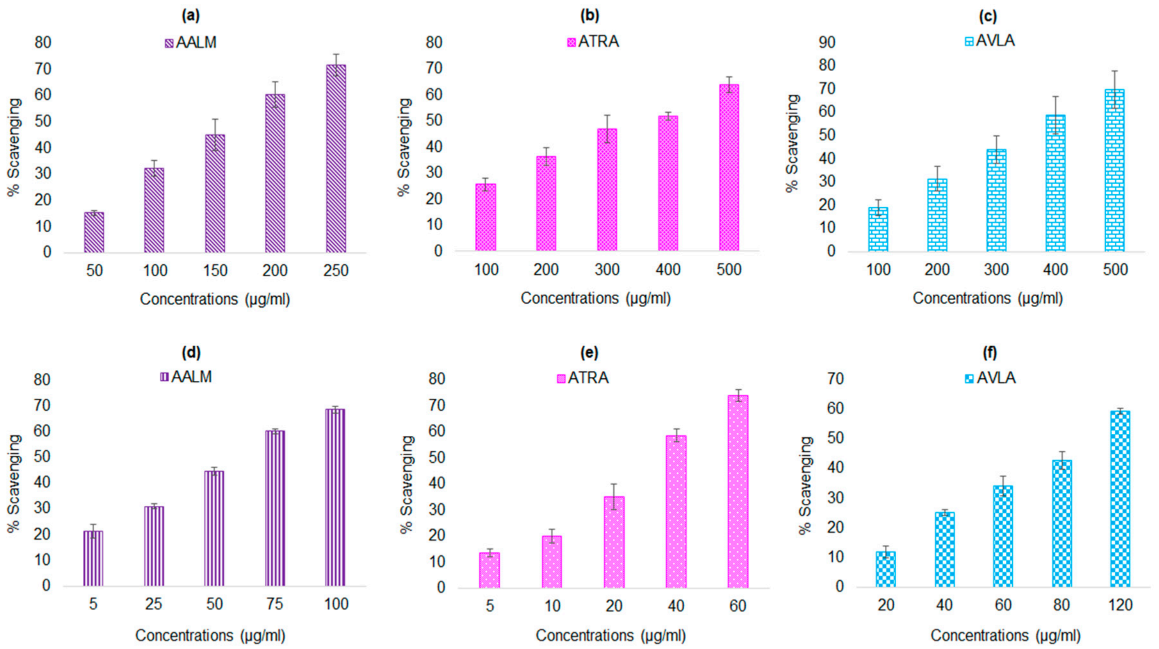

Figure S1. Bar graphs show concentration-dependent free radical scavenging. Concentration (µg/ml) vs. % scavenging by 3 potential extracts: In DPPH assay (**a**) AALM (AA leaves methanolic), (**b**) ATRA (AT roots acetone), and (**c**) AVLA (AV leaves acetone); In ABTS assay (**d**) AALM, (**e**) ATRA, and (**f**) AVLA.
